# MCPIP3 orchestrates the balance of epidermal proliferation and differentiation

**DOI:** 10.1186/s12964-025-02184-1

**Published:** 2025-04-08

**Authors:** Agata Lichawska-Cieslar, Weronika Szukala, Pawel Pilat, Leopold Eckhart, Jacek C Szepietowski, Jolanta Jura

**Affiliations:** 1 Department of General Biochemistry, Faculty of Biochemistry, Biophysics and Biotechnology, Jagiellonian University, Gronostajowa 7, Krakow, 30-387 Poland; 2 Doctoral School of Exact and Natural Sciences, Jagiellonian University, Lojasiewicza 11, Krakow, 30-348 Poland; 3 Department of Dermatology, Medical University of Vienna, Währinger Gurtel 18-20, Vienna, 1090 Austria; 4 Faculty of Medicine, Wroclaw University of Science and Technology, Grunwaldzki sq. 11, Wroclaw, 51-377 Poland

**Keywords:** MCPIP3, Keratinocytes, Skin, Inflammation, Proliferation, Differentiation, Psoriasis

## Abstract

**Background:**

Monocyte chemoattractant protein-induced protein 3 (MCPIP3), also called Regnase-3 and encoded by the *ZC3H12C* gene, is a member of the MCPIP family of RNases. Previous studies showed that MCPIP1 in keratinocytes plays a pivotal role in the maintenance of skin integrity and immunological function. Given that the expression of MCPIP3, similar to that of MCPIP1, is increased in psoriatic lesions compared with uninvolved skin, a role of MCPIP3 in the regulation of keratinocyte and epidermal biology was hypothesized.

**Methods:**

This study aimed to investigate the specific function of the MCPIP3 protein in the skin. The expression pattern of MCPIP3 was studied in normal human epidermal keratinocytes (NHEKs) subjected to in vitro differentiation and upon stimulation with proinflammatory factors. Mice with keratinocyte-specific deletion of MCPIP3 (Mcpip3^loxP/loxP^Krt14^Cre^; MCPIP3^EKO^) were generated and characterized. The response of the skin of MCPIP3^EKO^ mice to imiquimod (IMQ) and 12-O-tetradecanoylphorbol-13-acetate (TPA) was investigated. The expression levels of key modulators of keratinocyte proliferation and differentiation were measured in MCPIP3^EKO^ model mice and in NHEKs transiently transfected with MCPIP3-specific siRNA. Reporter assays were used to identify direct targets of MCPIP3 nucleolytic activity.

**Results:**

In human keratinocytes, the expression of *ZC3H12C*/MCPIP3 was rapidly induced by stimulation with TPA, IL-17a, IL-36α, and TNF-α. Although mice with keratinocyte-specific deletion of MCPIP3 (MCPIP3^EKO^) did not develop skin inflammation, they displayed abnormalities in skin morphology. Stimulation with IMQ and TPA exacerbated epidermal hyperplasia caused by keratinocyte-specific deficiency of MCPIP3 and led to abnormal epidermal differentiation. The expression levels of keratinocyte proliferation and differentiation markers, such as keratin-14, cyclin B1, involucrin, and the S100 calcium-binding proteins S100A7/A9, were increased in NHEKs in which MCPIP3 expression was silenced. MCPIP3 negatively regulates the level of cyclin B1 mRNA *via* direct nucleolytic cleavage within its 3’ untranslated region.

**Conclusions:**

The MCPIP3 protein modulates the balance of keratinocyte proliferation and differentiation and functions as a regulator of epidermal morphology in vivo.

**Supplementary Information:**

The online version contains supplementary material available at 10.1186/s12964-025-02184-1.

## Background

The skin is the largest self-renewing organ of the human body and provides an interface between the organism and the environment [1, 2]. Keratinocytes are the major cell type of the epidermis, the outermost layer of the skin. The epidermis consists of basal, spinous, granular and cornified strata [2]. Its formation depends on continuous and coordinated processes of keratinocyte proliferation and differentiation. At each stage of differentiation, keratinocytes express specific structural proteins, such as keratins (Krt) and lipids. In particular, the expression of Krt14 and Krt5 is restricted to the basal layer; Krt10 and Krt1 are expressed in the more differentiated suprabasal layer; and highly differentiated cells are characterized by loricrin, involucrin, filaggrin, SPRR and S100A expression [3, 4]. The terminal differentiation program is orchestrated by numerous cytokines and chemokines, but its main trigger is the calcium gradient in the epidermis [3]. Any disturbances in the keratinocyte differentiation program result in weakened barrier function [1]. When the protective barrier of the skin becomes dysfunctional, several types of inflammatory disorders may develop. Chronic inflammatory skin disorders, such as psoriasis or atopic dermatitis, are characterized by hyperproliferation of keratinocytes and/or their dysfunctional differentiation [5, 6].

Monocyte chemoattractant protein 3 (MCPIP3; encoded by the *ZC3H12C* gene) is a member of the MCPIP family. The common feature of these proteins is the presence of the PilT N-terminus (PIN-like) domain, which possesses enzymatic activity, and a CCCH-type zinc finger domain, which is required for interaction with nucleic acids [7, 8]. MCPIP1 (Regnase-1; encoded by the *ZC3H12A* gene) is the best-studied MCPIP family member. Mechanistically, MCPIP1 binds to the stem‒loop structures present in 3’ untranslated regions (UTRs) and mediates the degradation of transcripts encoding proinflammatory cytokines (e.g. IL-6 and IL-1β) [9–11]. Although the MCPIP1 and MCPIP3 proteins share similar domain structures, many fewer studies have been conducted on MCPIP3. In contrast to *Zc3h12a*^*−/−*^ knockout mice, *Zc3h12c*^*−/−*^ mice do not develop systemic autoimmunity but show elevated IFN signaling [10, 12]. Like MCPIP1, MCPIP3 is a negative regulator of NF-κB signaling and attenuates inflammation in endothelial cells [13, 14]. In mouse primary macrophages, MCPIP1 and MCPIP3 are similarly induced by TLR agonists and cytokines [8, 12]. The MCPIP3 RNase also binds the 3’UTR sequence. Reporter assays revealed that the 3’UTRs of *ZC3H12A*, *IL6* (similar to MCPIP1) and *TNFA* but not of *IL1B* (unlike MCPIP1) are elements that are responsive to MCPIP3 [12, 13, 15, 16].

The role of MCPIP1 in keratinocyte biology has recently been reported. We previously showed that the loss of murine MCPIP1 results in the upregulation of transcripts related to keratinocyte differentiation and the immune response. We further showed that Mcpip1^EKO^ (epidermal knockout) mice progressively develop skin inflammation [17]. In the human psoriatic epidermis, MCPIP1 is activated transcriptionally and translationally [18]. MCPIP1 deficiency contributes to increased sensitivity to imiquimod (IMQ) [18–21]. Interestingly, in genome-wide association studies, a polymorphism in the *ZC3H12C* gene was associated with psoriasis, suggesting the potential involvement of MCPIP3 in the pathogenesis of this disease [22]. Further analyses of psoriatic patient tissue biopsies revealed that the transcription of *ZC3H12C* is greater in psoriatic lesions than in uninvolved skin [15, 23]. However, in an experimental model of psoriasiform inflammation in mice, whole-organ deficiency of MCPIP3 did not improve the outcome of IMQ-induced inflammation. One of the proposed mechanisms involves the protective role of intradermal IL-6 secreted by MCPIP3-deficient plasmacytoid dendritic cells [16, 23]. However, the effects of keratinocyte MCPIP3 on the development of psoriasis and other skin diseases with an inflammatory background remain unknown.

This study aimed to investigate the specific role of the MCPIP3 RNase in skin biology. We used complementary in vitro and in vivo approaches to determine how MCPIP3 regulates keratinocyte and epidermal function.

## Methods

### Cell culture

Normal human epidermal keratinocytes (NHEKs) were obtained from Lonza and cultured in keratinocyte basal medium (KBM-Gold) supplemented with the KGM-Gold Bullet Kit (Lonza Group, Ltd., Basel, Switzerland). HEK293 cells (ATCC; Manassas, VA, USA) and human immortalized HaCaT keratinocytes [24] were cultured in high-glucose DMEM supplemented with 10% fetal bovine serum (FBS; Sigma‒Aldrich, Saint-Louis, MO, USA). A431 cells (ATTC) with doxycycline-inducible overexpression of MCPIP1 were used as described previously [25]. All the cells were grown at 37 °C in a 5% CO_2_ humidified atmosphere.

### In vitro differentiation, stimulation and transfection of NHEKs

For in vitro differentiation, NHEKs seeded at 50% confluency were analyzed 24 h (day 0) or 7 days (day 6) after seeding. For calcium-dependent differentiation, 1.8 mM CaCl_2_ was added to the medium on day 0, and the samples were analyzed on days 0, 3 or 6. The medium was changed every 2 days. For stimulation, NHEKs were treated with 2.5 ng/ml 12-*O*-tetradecanoylphorbol-13-acetate (TPA; Sigma‒Aldrich), 100 ng/ml human recombinant IL-17a (R&D, Abingdon, UK), 100 ng/ml human recombinant IL-36α (R&D), 100 ng/ml human recombinant TNF-α (R&D), 100 ng/ml human recombinant IL-6 (Cell Signaling Technology, Danvers, MA, USA) or 100 ng/ml human recombinant IFN-γ (Strathmann Biotech AG, Hamburg, Germany). For silencing of MCPIP3, NHEKs were transfected with 20 nM MCPIP3-specific (siGENOME human ZC3H12C SMARTpool) or nontargeting control siRNA (Non-Targeting siRNA Pool#1; Dharmacon) using jetPRIME Reagent (Promega, Madison, USA) for 48–72 h.

### Cell viability assay

HaCaT cells were seeded in triplicates at a density of 1.5 × 10^3^ cells/well in 96-well plates and treated with siRNA 24 h after seeding. Cell viability was measured via a colorimetric Thiazolyl Blue Tetrazolium Blue (MTT; Sigma-Aldrich) assay. The absorbance was measured at 570 nm, with a reference wavelength of 650 nm on Synergy H1 microplate reader (BioTeK, WA, USA).

### Mice

Mice with loxP-flanked sites in exon 2 of MCPIP3 (ENSMUST00000165519.1) were designed and generated by GemPharmatech Co., Ltd. (Nanjing, China) through targeted gene modification using CRISPR/Cas9 technology. We subsequently crossed these mice (Zc3h12c^loxP/loxP^) with Krt14Cre^tg+^ mice [26] to obtain keratinocyte-specific Mcpip3 knockout (Krt14Cre^tg+^*Zc3h12c*^*loxP/loxP*^; herein, MCPIP3^EKO^ mice). All the mice were bred on a C57BL/6 background. For genotyping, DNA was extracted from tail tissues using the KAPA Mouse Genotyping Kit (KAPA Biosystems, Woburn, MA, USA). The primer sequences (Sigma‒Aldrich) are listed in Table S1.

### Mouse primary keratinocyte (MPK) isolation

MPKs were isolated from newborn (0–2 days) control or Mcpip3^EKO^ pups as previously described [17]. The epidermis was separated from the dermis by overnight incubation with Dispase II (Roche, Basel, Switzerland). Primary keratinocytes were cultured in CnT-Prime Epithelial Cell Culture Medium (CELLnTEC, Bern, Switzerland) and collected after 48 h for RNA/protein isolation.

### IMQ skin treatment

For the IMQ-induced model of psoriasis-like skin inflammation, we applied a daily topical dose of 62.5 mg of commercially available IMQ cream (5%) (Aldara; 3 M Pharmaceuticals, St. Paul, MN, USA) or Vaseline (as a control for the experiment) to the shaved backs of 6- to 8-week-old mice for 6 days, as previously described [27]. The clinical Psoriasis Area and Severity Index (PASI) was used to indicate the severity of inflammation by scoring redness, scaling and thickness on a scale from 0 to 4. Skin thickness was measured daily with a digital micrometer. The mice were sacrificed after 2 or 6 days of treatment, and skin specimens were collected for further analysis.

### TPA skin treatment

For TPA treatment, 6- to 8-week-old mice were shaved, and the next day, the mice were treated with 15 µg of TPA (Sigma‒Aldrich) reconstituted in 200 µl of acetone or with acetone alone (as a control for the experiment). The treatment was repeated 3 days later. The mice were sacrificed 6 days after the first TPA treatment, and skin samples were collected for further analyses.

### RNA isolation and RT‒qPCR analysis

The collected mouse skin samples were snap-frozen in liquid nitrogen and stored at -80 °C for downstream analysis. For RNA isolation, the samples were homogenized in Fenozol (A&A Biotechnology, Gdansk, Polska) using a tissue homogenizer (OMNI International, Kennesaw, GA, USA). Cellular RNA was also isolated using Fenozol. cDNA was synthesized from 1 µg of RNA using M-MLV reverse transcriptase (Promega), and quantitative real-time PCR was performed with SYBR Green Master Mix (A&A Biotechnology) and a QuantStudio3 thermocycler (Thermo Fisher Scientific). The sequences of the primers used are listed in Table S1.

### RNA sequencing (RNA-seq)

The RNA samples were subjected to DNase I treatment and concentrated *via* RNA Clean and Concentrator-5 (Zymo Research, Irvine, CA, USA*).* RNA-seq was performed by CeGaT GmbH (Tübingen, Germany) on a NovaSeq 6000 instrument. One hundred nanograms of total RNA was used for library preparation using TruSeq Stranded mRNA (Illumina, San Diego, CA, USA). The sequencing reads were multiplexed with Illumina bcl2fastq (2.20), adapters were trimmed with Skewer (version 0.2.2) [28], and then, the raw reads were aligned to mm10 using STAR (version 2.7.3) [29]. The quality of the FASTQ files was analyzed with FastQC (version 0.11.5) [30]. Differential expression analysis between groups was performed with DESeq2 (version 1.24.0) [31] in R (version 4.0.4). The functional annotation of differentially expressed genes (DEGs) was performed using the R package ClusterProfiler (version 4.4) [32]. Volcano plots and dot plots were created using the ggplot2 libraries in R.

### Histological and Immunofluorescence staining

Murine skin samples were fixed in 4% buffered formalin overnight, processed for dehydration and embedded in paraffin. Then, 5–8 μm sections were cut *via* a microtome (Leica Microsystems, Wetzlar, Germany) and stained with hematoxylin and eosin (Sigma‒Aldrich) *via* a standard procedure. For immunofluorescence staining, antigen retrieval was performed by a 30-min incubation at 95 °C in 10 mM citrate buffer (pH 6.0). The sections were subsequently blocked for 1 h with 5% horse serum containing 1% BSA and 0.2% Triton X-100 (BioShop, Burlington, Canada) in PBS and incubated with primary antibodies overnight at 4 °C. The next day, the slides were incubated with secondary antibodies and Hoechst 33,258 (Thermo Fisher Scientific, Waltham, MA, USA) for 1 h at room temperature, rinsed with PBS and mounted with fluorescent mounting medium (Dako). The samples were visualized under a Leica DMC5400 fluorescence microscope (Leica Microsystems, Wetzlar, Germany). All figures were prepared using ImageJ [33]. The antibodies used in this study are listed in Table S2.

### Western blot analysis

For protein isolation, the cells were lysed in RIPA buffer supplemented with Complete Protease Inhibitor Cocktail (Roche) and PhosSTOP Phosphatase Inhibitor Cocktail (Roche). The protein concentration was measured with a bicinchoninic acid assay. SDS‒PAGE analysis, transfer to a PVDF membrane and signal detection were performed as described previously [17]. The antibodies used in this study are listed in Table S2. Densitometric analysis was performed using the ImageJ software.

### Luciferase assay

Fragments containing the 3’UTR sequences of human *CCNB1*, *KRT14*,* CLCA2* and *SPRR2D* were amplified using gene-specific primers (Table S1) and inserted into the pmirGLO dual-luciferase plasmid (FJ376737; Promega, Madison, WI, USA) *via* the NheI and SalI sites. All the constructs were verified by sequencing. HEK293 cells were seeded on 48-well plates one day before cotransfection with 282 ng pmirGLO-3’UTR or pmirGLO-empty plasmid together with 18 ng of pcDNA3.0 empty or pcDNA3.0 encoding MCPIP3 or a catalytically inactive D251N mutant using jetPRIME Reagent (Promega). Twenty-four hours later, the cells were lysed, and the supernatants were analyzed using the Dual-Luciferase Reporter Assay System (Promega) on a Turner Biosystems 20/20n luminometer (Promega). Each sample’s luciferase activity was normalized to the luciferase activity of the corresponding pmirGLO-empty vector.

### Statistical analysis

Statistical analyses were performed using GraphPad Prism 8 (GraphPad, La Jolla, CA, USA). All the figures were created using CorelDraw 2021 (Corel Corporation, ON, Canada).

## Results

### Stimulation of keratinocytes with TPA, IL-17a, IL-36α, and TNF-α upregulates the expression of ZC3H12C/MCPIP3

**Fig. 1:**
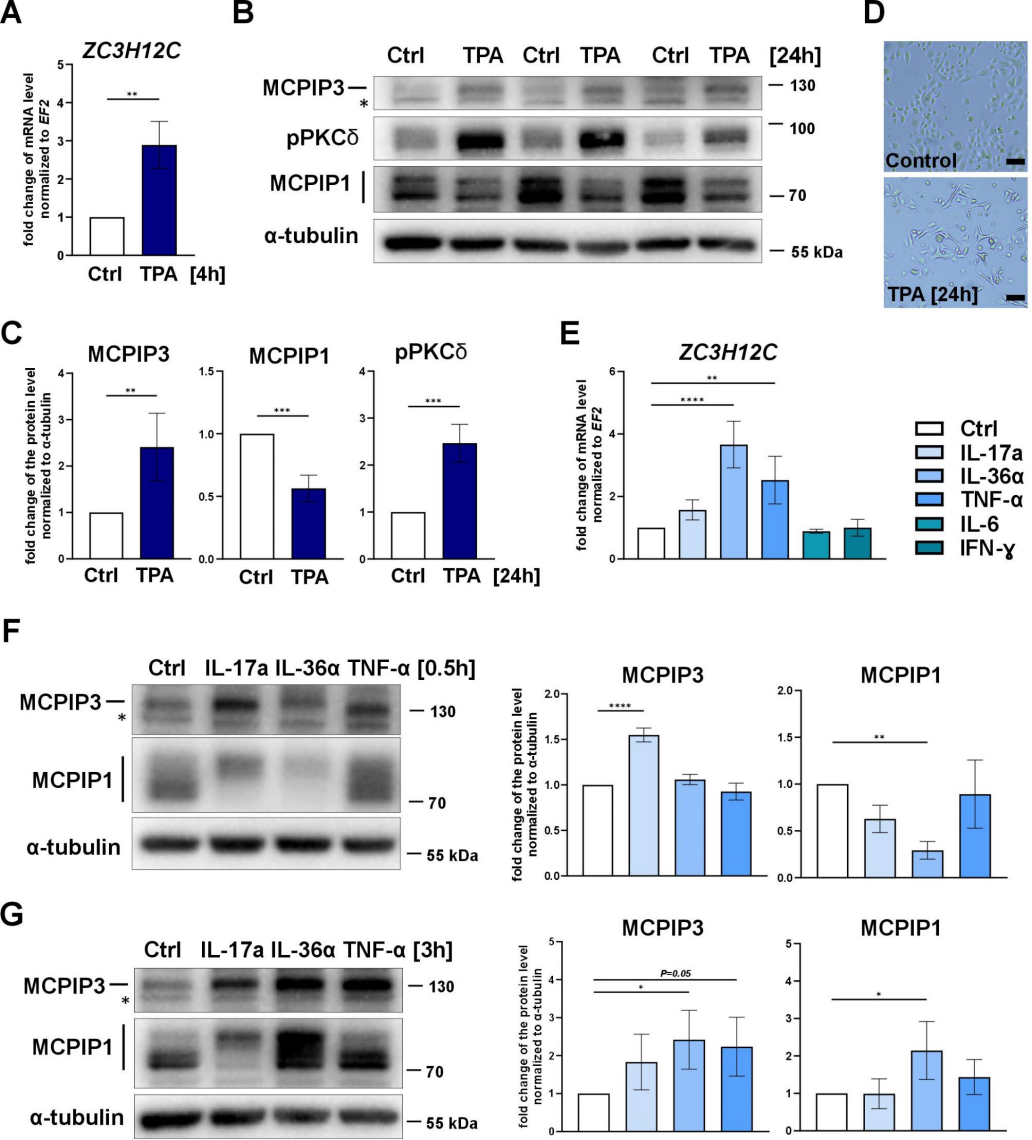
Expression profiles of MCPIP1 and MCPIP3 in human keratinocytes. **(A)** RT‒qPCR analysis of *ZC3H12C* transcript levels in NHEKs treated with TPA for 4 h (*n* = 3). **(B)** Western blot analysis of MCPIP1, MCPIP3, p-PKCδ (Thr505) and α-tubulin in NHEKs treated with TPA for 24 h. **(C)** Densitometric analysis of protein bands from Western blot analysis (*n* = 4). **(D)** Bright field images of NHEKs treated with TPA for 24 h. **(E)** RT‒qPCR analysis of *ZC3H12C* transcript levels in NHEKs treated with IL-17a, IL-36α, TNF-α, IL-6, or IFN-γ for 3 h (*n* = 3). **(F-G)** Western blot analysis of MCPIP1, MCPIP3 and α-tubulin in NHEKs treated with IL-17a, IL-36α, and TNF-α for 0.5 h (F) or 3 h (G). The graphs show the results of the densitometric analysis (*n* = 3–4). The data are shown as the means ± standard deviations. *EF2* was used as a reference gene (A, E). * indicates an unspecific band (B, F, G). Unpaired t tests (A, C) or one-way ANOVA (E, F, G) was used for statistical analysis; * *p* < 0.05, ** *p* < 0.01, *** *p* < 0.001, and **** *p* < 0.0001

To analyze the role of MCPIP3, we investigated its expression pattern in NHEKs. First, the level of MCPIP3 was investigated in NHEKs during in vitro differentiation. NHEKs were grown in the presence or absence of calcium for 6 days (Fig. S1A). As expected, both models, confluency-induced and calcium-driven models, showed in a significant increase in KRT10 levels and the activation of PKCα/β kinase. Consistent with previous reports [17], the expression of MCPIP1 increased in confluent, differentiated keratinocytes. In contrast, the protein level of MCPIP3 was not significantly altered on day 6 of differentiation in either model. Interestingly, an initial ~ 50% reduction in its level was observed on day 3 (Fig. S1B-C). At the transcript level, differentiated keratinocytes presented a significant reduction in *ZC3H12C* mRNA levels (Fig. S1D). Previous report indicated that MCPIP1 negatively regulates the *ZC3H12C* mRNA half-life [15]. In agreement with this observation, we found that the level of *ZC3H12C* mRNA was downregulated in cells overexpressing wild type MCPIP1, but not its inactive mutant (Fig. S1E). Thus, high expression of MCPIP1 in differentiated keratinocytes in vitro is the most plausible factor promoting *ZC3H12C* mRNA degradation in these models. Next, we investigated whether the expression of MCPIP3 is modulated upon treatment of NHEKs with a stimulant that affects the balance of keratinocyte proliferation and differentiation. We found that treatment with TPA, a tumor-promoting phorbol ester, significantly increased the MCPIP3 transcript and protein levels (Fig. 1A-C). As expected, stimulation with TPA for 24 h induced significant activation of the PKCδ isoform and morphologically induced some obvious changes in NHEKs, suggesting a switch toward squamous differentiation (Fig. 1D). These changes correlated with altered expression of genes related to proliferation (decreased level of *KRT14*) and terminal differentiation (increased levels of *IVL* and *SPRR2D*) (Fig. S1F). Overall, the expression profiles of MCPIP1 and MCPIP3 in all the models of differentiating keratinocytes were different.

Previous studies have indicated that MCPIP3 is transcriptionally activated in psoriatic skin, suggesting that it may play a role in the pathogenesis of skin disorders with an inflammatory background [15, 22, 23]. We analyzed the expression pattern of MCPIP3 in NHEKs stimulated with various cytokines, mostly related to psoriatic disease, and found that the *ZC3H12C* mRNA and MCPIP3 protein levels were rapidly increased following treatment with IL-17a, IL-36α, and TNF-α (Fig. 1E-G).

### Mice with keratinocyte-specific loss of MCPIP3 develop epidermal hyperplasia

**Fig. 2:**
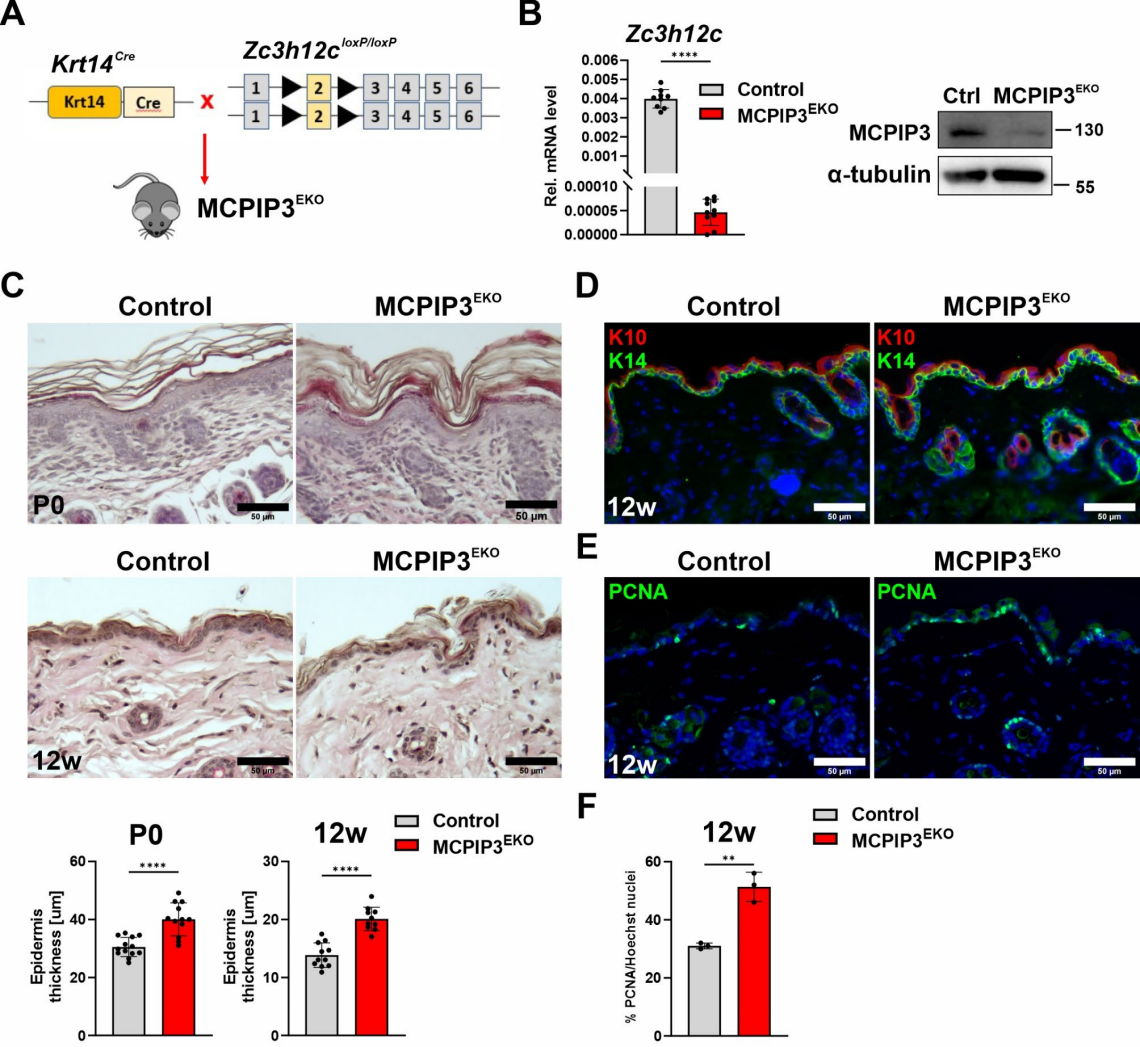
Keratinocyte-specific loss of MCPIP3 leads to increased epidermal thickness. **(A)** Scheme illustrating the generation of mice with keratinocyte-specific loss of MCPIP3. **(B) Left panel**: RT‒qPCR analysis of *Zc3h12c* expression in MPKs from neonatal skin (*n* = 9–10). *Ef2* was used as a reference gene. **Right panel**: Representative Western blot analysis of MCPIP3 and α-tubulin in neonatal MPKs. **(C)** H&E staining and quantification of the epidermal thickness of neonatal skin and that of the 12-week-old mice (*n* = 12–13 for neonatal skin and *n* = 10–11 for 12-week-old mice). **(D)** Immunofluorescence staining for KRT10 (K10) and KRT14 (K14). **(E)** Immunofluorescence staining for PCNA. **(F)** Quantification of the percentage of the PCNA-positive cells (*n*=3). Data are shown as the mean ± standard deviation. Unpaired t tests were used for statistical analysis; ** *p* < 0.01; **** *p* < 0.0001. Scale bar = 50 μm

To evaluate the in vivo role of MCPIP3 in keratinocytes, we generated conditional knockout mice by crossing the floxed strain with transgenic mice expressing Cre recombinase under the keratin-14 promoter (Fig. 2A). This strategy resulted in epidermis-specific deletion of the targeted region in Zc3h12c^loxP/loxP^Krt14Cre mice, herein referred to as MCPIP3^EKO^. We verified a significant reduction in *Zc3h12c* mRNA and MCPIP3 protein expression in MCPIP3^EKO^ primary neonatal keratinocytes, confirming the successful elimination of MCPIP3 (Fig. 2B). The MCPIP3^EKO^ pups were born without any obvious phenotypic abnormalities. However, as the histological analysis revealed, their epidermal thickness was significantly increased by ~ 1.3-fold (Fig. 2C). We previously demonstrated that aging mice with keratinocyte-specific deletion of MCPIP1 develop spontaneous skin and systemic inflammation [17]. However, in MCPIP3^EKO^ mice, no phenotypic abnormalities were observed (Fig. S2A). Furthermore, no heterogeneity in body weight or spleen or lymph node weights was detected between the Zc3h12c^loxP/loxP^ control and MCPIP3^EKO^ 12-week-old mice (Fig. S2B). Microscopic evaluation of H&E-stained skin sections from 12-week-old mice revealed a significant ~ 1.5-fold increase in epidermal thickness in the MCPIP3^EKO^ mice compared with their control littermates. We also detected increased stratification of the MCPIP3^EKO^ mouse epidermis, which was indicated by a more abundant and less dense stratified layer (Fig. 2C). Next, we analyzed the distribution of markers for keratinocyte proliferation and differentiation, KRT14 and KRT10, respectively, and the presence of PCNA-positive cells (Fig. 2D-E). We found that there were ~ 1.6 more PCNA + cells in the MCPIP3^EKO^ epidermis than in the control epidermis, confirming the increased proliferation rate (Fig. 2F). No signs of inflammation or inflammatory influx in the skin of the keratinocyte-specific MCPIP3 knockout mice were detected *via* H&E staining, RT‒qPCR analysis of the expression levels of some proinflammatory factors, or immunofluorescence analysis of F4/80 + infiltrates (Fig. 2C and S2C‒D).

### Loss of MCPIP3 function in murine keratinocytes leads to global changes in the skin transcriptome

**Fig. 3:**
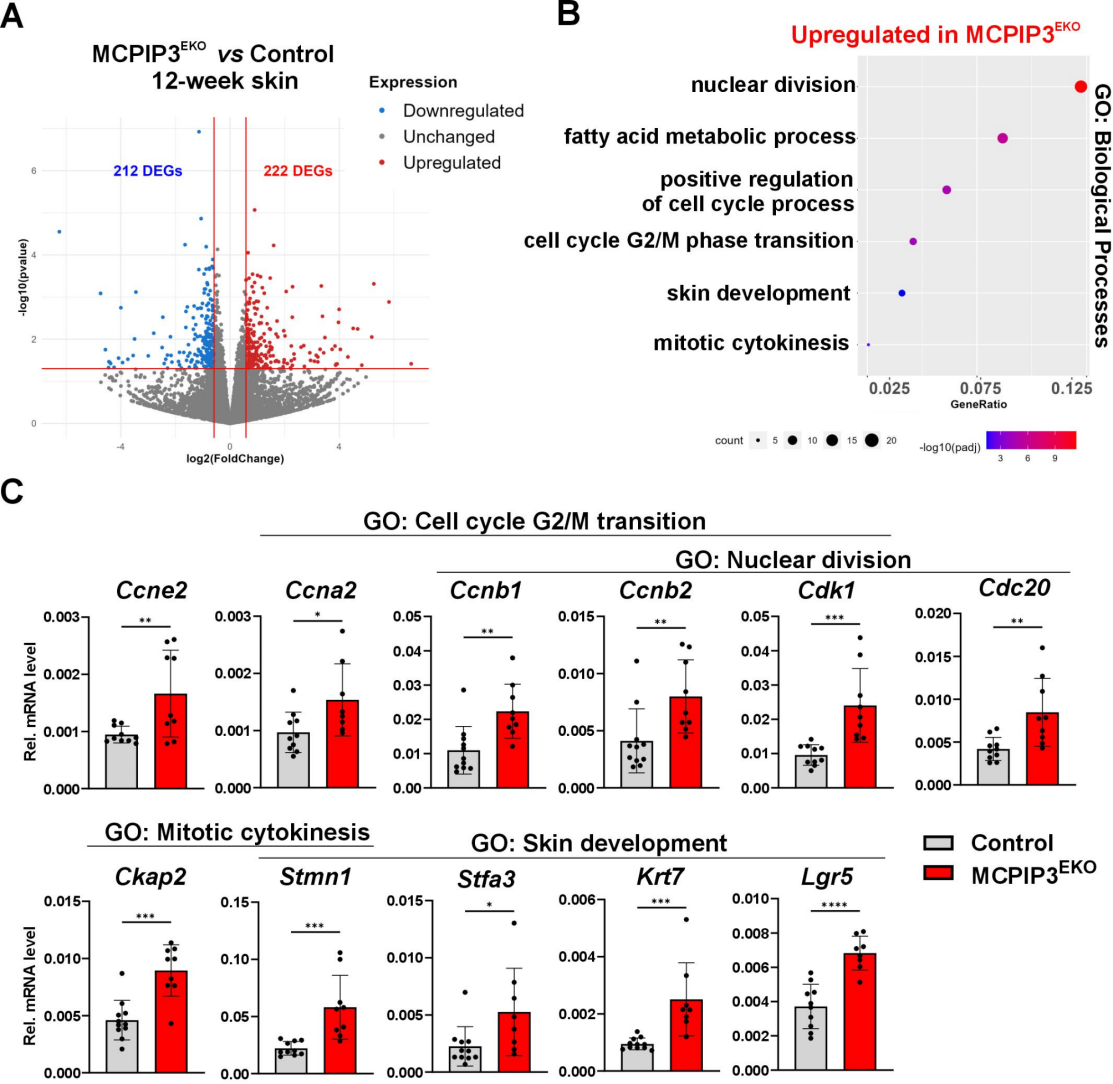
RNA-seq analysis of the skin of 12-week-old control and MCPIP3^EKO^ mice. **(A)** Volcano plot representing DEGs in the skin of 12-week-old MCPIP3^EKO^ mice compared with that of control mice. p value < 0.05; FC > 1.5. **(B)** GO enrichment analysis of selected upregulated biological processes in MCPIP3^EKO^ skin. **(C)** RT‒qPCR analysis of *Ccne2*, *Ccna2*, *Ccnb1*, *Ccnb2*, *Cdk1*, *Cdc20*, *Ckap2*, *Stmn1*, *Stfa3*, *Krt7* and *Lgr5* expression levels in skin (*n* = 8–11). *Ef2* was used as a reference gene. The data are shown as the means ± standard deviations. Unpaired t tests were used for statistical analysis; * *p* < 0.05, ** *p* < 0.01, *** *p* < 0.001 and **** *p* < 0.0001

Total RNA was isolated from the skin of four control and four MCPIP3^EKO^ 12-week-old mice. Global sequencing of coding RNA was then performed. We identified 434 DEGs, 222 of which were upregulated in MCPIP3^EKO^ skin (Fig. 3A). Gene Ontology enrichment analysis revealed that these genes were mostly functionally assigned to biological processes related to cell cycle progression, particularly ‘nuclear division’, ‘regulation of the cell cycle’, ‘G2/M phase transition’ and ‘mitotic cytokinesis’. We also identified DEGs related to fatty acid metabolic processes and skin development (Fig. 3B). The differential gene expression patterns were subsequently validated by quantitative real-time PCR on a larger number of samples. In particular, the mRNA levels of a plethora of S- and G2/M-phase cyclins, including cyclin E2, cyclin A2, cyclin B1 and cyclin B2, along with the key G2/M kinase Cdk1 and an essential modulator of mitotic exit, the Cdc20 protein, were significantly increased in MCPIP3^EKO^ skin. The transcription of genes encoding critical modulators of microtubule polymerization and cytokinesis, CKAP2 and stathmin, was increased. We also observed elevated expression levels of genes related to keratinocyte differentiation and skin development, such as *Stfa3*, *Krt7* and *Lgr5* (Fig. 3C).

### TPA and IMQ exacerbate epidermal hyperplasia and abnormal epidermal differentiation caused by keratinocyte-specific deficiency of MCPIP3

**Fig. 4:**
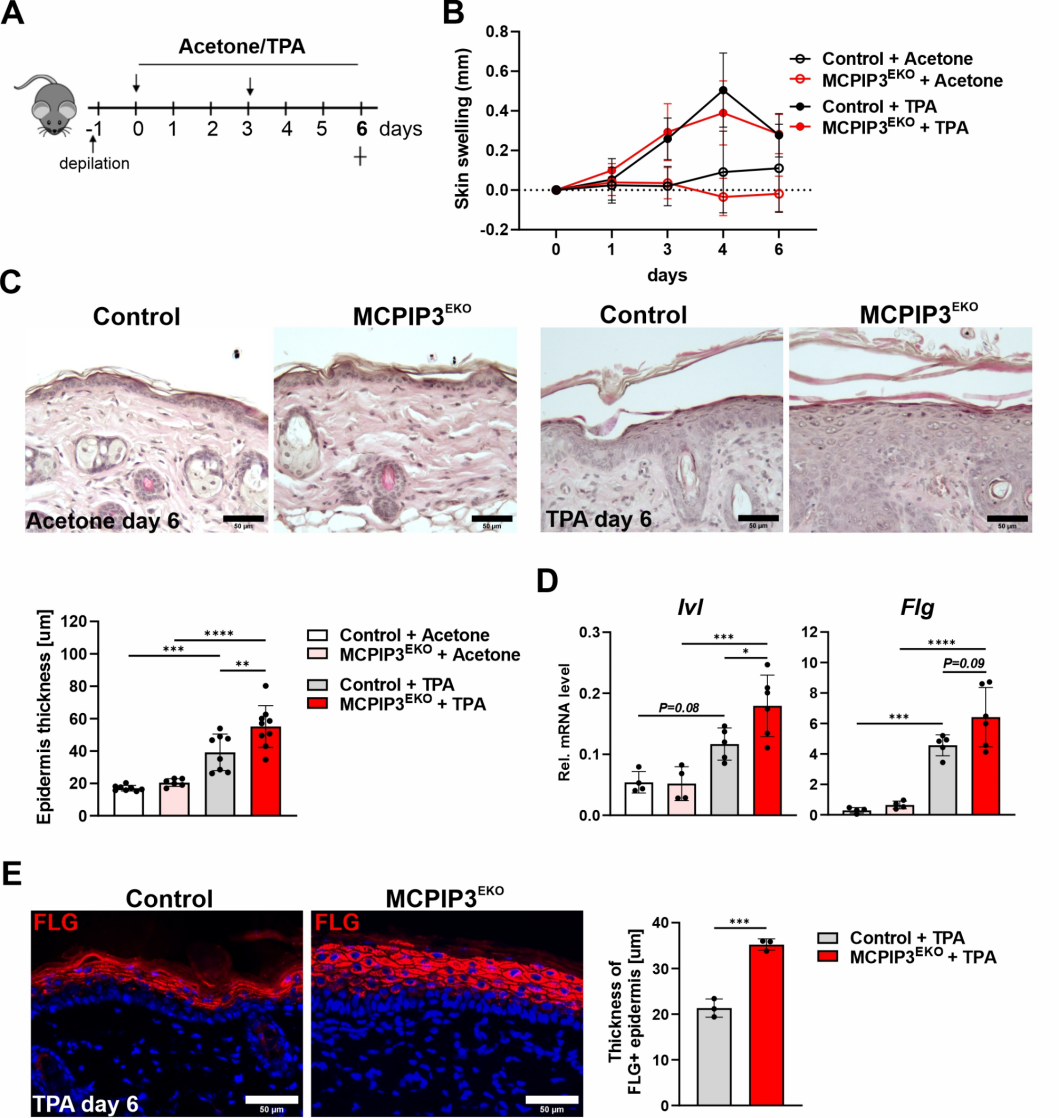
TPA treatment exacerbates MCPIP3 ^EKO^ epidermal hyperproliferation. **(A)** Graphical timeline of TPA treatment. **(B)** Skin swelling in the TPA- or acetone-treated back skin of control and MCPIP3^EKO^ mice (*n* = 4–5 for acetone and *n* = 5–6 for TPA). **(C)** H&E staining and quantification of epidermal thickness in mouse skin on day 6 (*n*=6‒9). **(D)** RT‒qPCR analysis of *Ivl* and *Flg* expression levels in control and MCPIP3^EKO^ skin treated with acetone or TPA for 6 days (*n* = 4‒6). *Ef2* was used as a reference gene. **(E)** Immunofluorescence staining for filaggrin (FLG) in TPA-treated skin. The graph shows the quantification of the thickness of the FLG + epidermis (*n*=3). The data are shown as the means ± standard deviations. One-way ANOVA (C-D) or unpaired t test (E) was used for statistical analysis; *p < 0.05, ** *p* < 0.01, *** *p* < 0.001 and ****p < 0.0001. Scale bar = 50 μm

We next analyzed the skin response of MCPIP3^EKO^ mice to TPA- and IMQ-induced inflammation. TPA was applied topically on the back skin (Fig. 4A). As expected, this treatment resulted in localized skin inflammation, but the mean degree of dorsal skin swelling was comparable between the MCPIP3^EKO^ and control mice (Fig. 4B). However, histological analysis revealed that 6 days of stimulation with TPA resulted in more pronounced and significantly increased epidermal hyperplasia in the MCPIP3^EKO^ mice. H&E staining also revealed more pronounced hyperkeratosis (Fig. 4C). Accordingly, TPA-treated MCPIP3^EKO^ skin was characterized by increased transcription of the *Ivl* and *Flg* genes (Fig. 4D). Immunofluorescence analysis revealed increased thickness of the FLG + layer in the MCPIP3 epidermal-knockout mice and more abundant areas of parakeratosis (Fig. 4E).

**Fig. 5:**
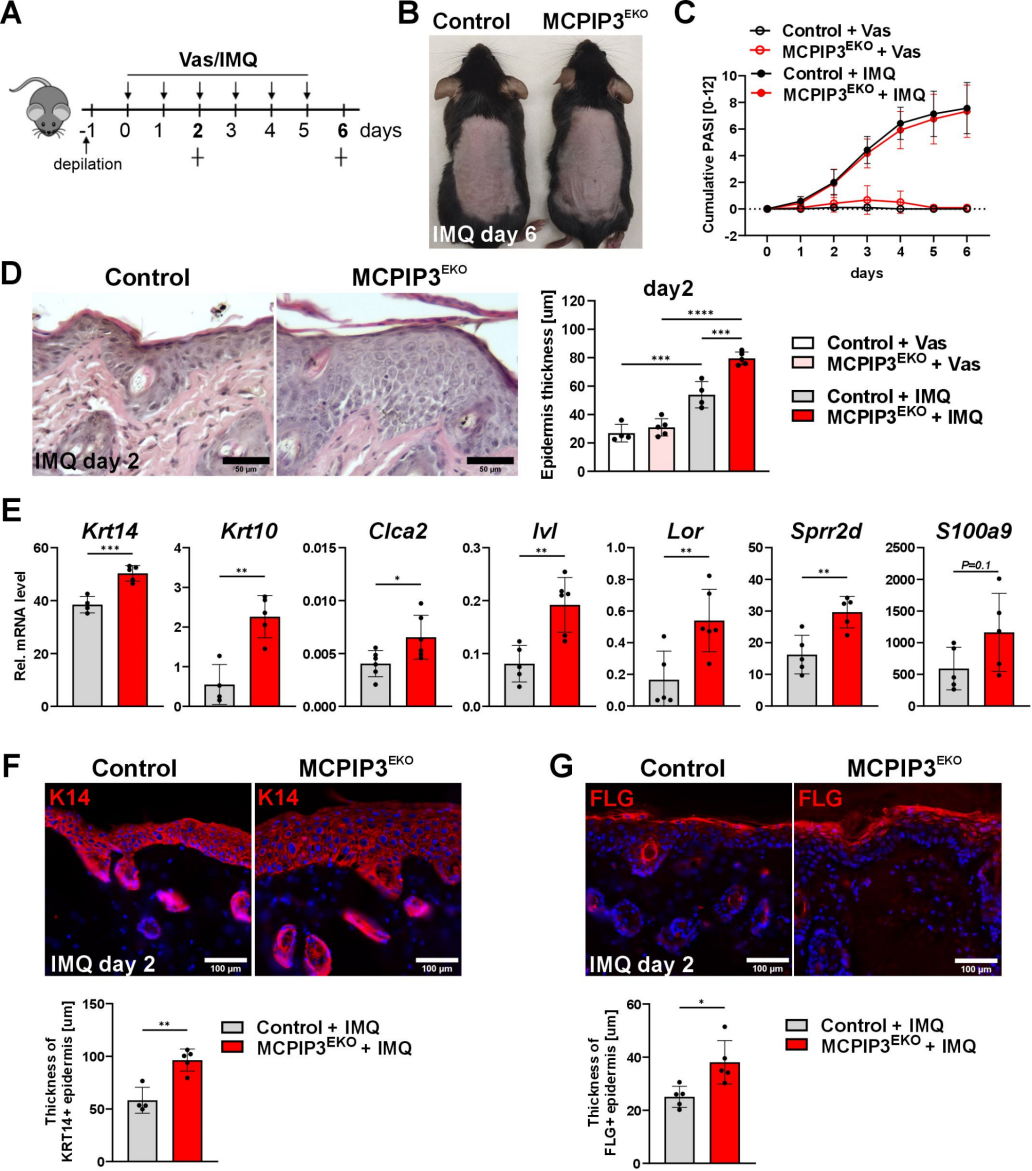
Deficiency of MCPIP3 alters the expression patterns of differentiation markers in psoriasis-like skin disease. (A) Graphical timeline of IMQ treatment. (B) Macroscopic appearance of mouse back skin on day 6. (C) Cumulative PASI score (erythema + scaling + thickness) over the 6 days of treatment (n=5-7). (D) H&E staining and quantification of epidermal thickness in mouse skin on day 2 (n=4-5). (E) RT‒qPCR analysis of *Krt14*, *Krt10*, *Clca2*, *Ivl*, *Lor*, *Sprr2d* and *S100a9* expression levels in control and MCPIP3^EKO^ skin treated with IMQ for 2 days (n=4‒6). *Ef2* was used as a reference gene. (F) Immunofluorescence staining for KRT14 (K14) in IMQ-treated skin on day 2. The graph shows the quantification of the thickness of the KRT14+ epidermis (n=4‒5). (G) Immunofluorescence staining for FLG in IMQ-treated skin on day 2. The graph shows the quantification of the thickness of the FLG+ epidermis (n=5). The data are shown as the means ± standard deviations. One-way ANOVA (D) or unpaired t tests (E, F, G) was used for statistical analysis; * *p* < 0.05, ** *p* < 0.01, *** *p* < 0.001 and **** *p* < 0.0001. Scale bar=100 µm

For the IMQ-induced skin inflammation model, the mice were subjected to stimulation with IMQ for 6 consecutive days as described by van der Fits (Fig. 5A) [27]. IMQ treatment led to a progressive increase in skin thickness, redness and scaling, but the cumulative PASI score suggested a similar response in the control and MCPIP3^EKO^ mice (Figs. 5B-C and S3A). The transcriptional activation of genes encoding major psoriatic-related factors, e.g., IL-23/Th17, IL-36 cytokines and Lcn2, was not significantly different between the control and MCPIP3^EKO^ groups **(**Fig. S3B**)**. However, microscopic evaluation revealed that compared with the control mice, the MCPIP3^EKO^ mice presented gross IMQ-induced changes in epidermal morphology. We observed that after 2 days of IMQ treatment, MCPIP3^EKO^ skin was characterized by increased hyperplasia and areas of parakeratosis (Fig. 5D and Fig. S3C**)**. Epidermal hyperplasia was also more obvious in MCPIP3^EKO^ mice skin after 6 days of IMQ stimulation **(**Fig. S3C**)**. We next analyzed the expression of a panel of keratinocyte proliferation and differentiation markers in IMQ-treated skin via RT‒qPCR and immunofluorescence. We observed increased expression levels of KRT14, which was consistent with the observation of epidermal hyperplasia in MCPIP3^EKO^ mice. Moreover, the expression of genes encoding proteins involved in epidermal differentiation, particularly KRT10 and CLCA2, as well as components of the cornified layer, namely, SPRR2D, S100A9, involucrin, and loricrin, significantly increased. Immunofluorescence analysis confirmed a greater abundance of KRT-14- and filaggrin (FLG)-positive cells within the MCPIP3^EKO^ IMQ-treated epidermis (Fig. 5E-G).

### In NHEKs, MCPIP3 modulates the expression of markers of keratinocyte proliferation and differentiation

**Fig. 6:**
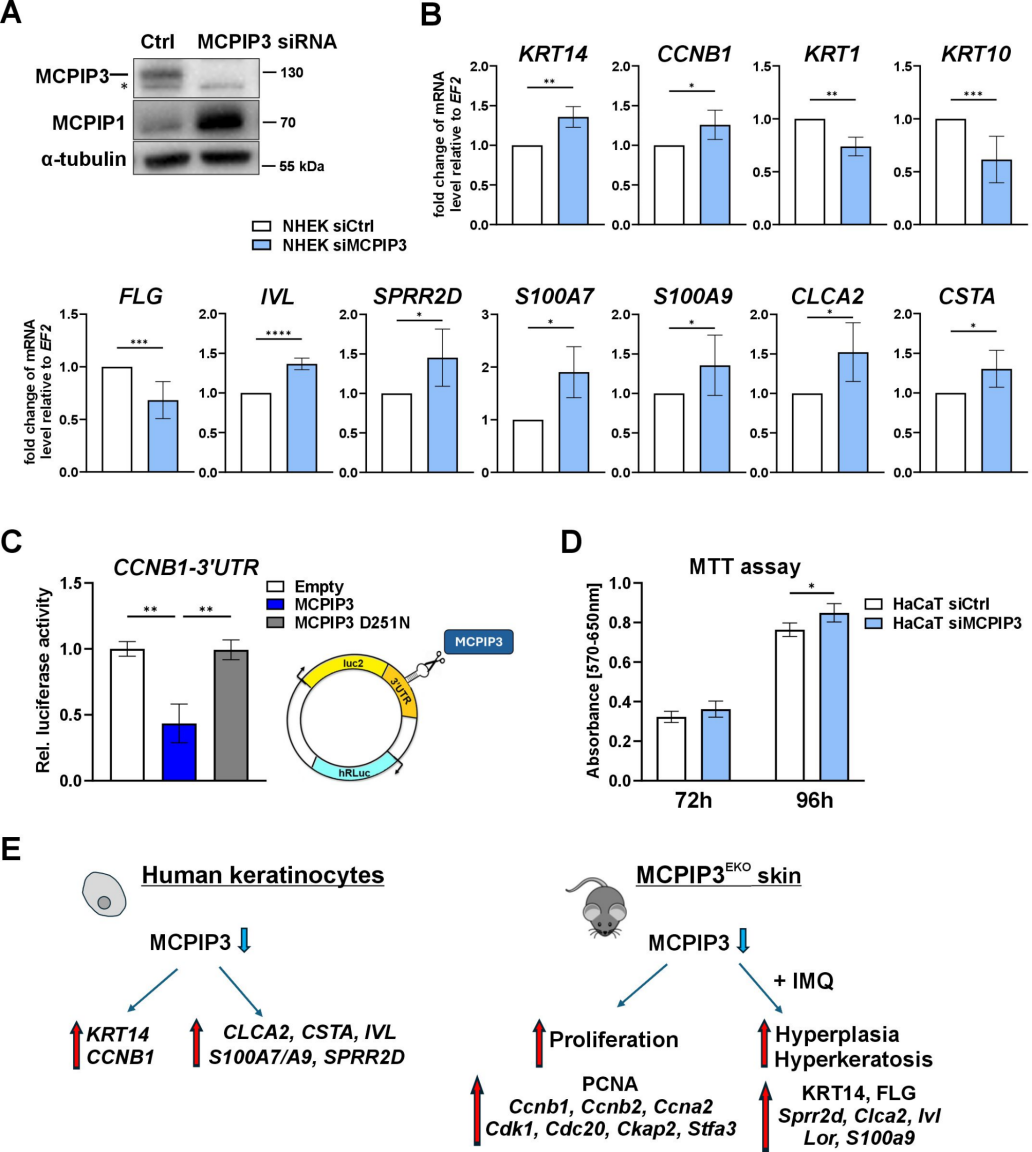
In human keratinocytes, MCPIP3 impairs the expression of genes related to proliferation and epidermal differentiation. **(A)** Representative Western blot analysis of MCPIP1, MCPIP3 and α-tubulin levels in NHEKs treated with control or MCPIP3 siRNA for 48 h; * indicates an unspecific band. **(B)** RT‒qPCR analysis of *KRT14*,* CCNB1*,* KRT1*,* KRT10*,* FLG*,* IVL*,* SPRR2D*,* S100A7*,* S100A9*,* CLCA2*, and *CSTA* expression levels. (*n* = 3–8). *EF2* was used as a reference gene. **(C)** HEK293 cells were cotransfected with a luciferase reporter pmirGLO plasmid containing the 3’UTR of human *CCNB1* and pcDNA3.0 (empty) or pcDNA3.0 encoding MCPIP3 or a catalytically inactive D251N mutant. The graph shows the calculated luciferase activity normalized to that of the pmirGLO-empty vector (*n* = 4). **(D)** The results of MTT viability test after 72–96 h treatment of HaCaT cells with control or MCPIP3-specific siRNA (*n* = 3–4). **(E)** Graphical representation of the results. The data are shown as the means ± standard deviations. (A). Unpaired t tests (B), one-way ANOVA (C) or two-way ANOVA (D) was used for statistical analysis; * *p* < 0.05, ** *p* < 0.01, *** *p* < 0.001, and ****p < 0.0001

To verify whether MCPIP3 plays a role specifically in human keratinocytes in modulating their proliferation and differentiation, we analyzed gene expression changes in NHEKs treated with MCPIP3-specific siRNA. Western blot analysis revealed effective downregulation of MCPIP3 protein expression in cells treated with gene-specific siRNAs for 48 h. As expected, downregulation of MCPIP3 increased the level of endogenous MCPIP1 protein (Fig. 6A). Silencing of MCPIP3 led to a significant increase in the transcript levels of genes related to keratinocyte proliferation, namely, *KRT14* and *CCBN1*, and a decrease in the levels of early differentiation markers and components of the granular layer, namely, *KRT1* and *KRT10.* The expression of *FLG*, encoding profilaggrin, a protein precursor present in the granule that is then cleaved and processed to filaggrin, was also decreased. Moreover, increased transcription of factors associated with the activation of terminal differentiation programs, such as involucrin, SPRR2D and S100A7/A9, as well as other factors related to differentiation, such as CLCA2 and cystatin (CSTA, a human homolog of mouse Stfa3), was observed (Fig. 6B). We verified that in keratinocytes stimulated with IL-36α, silencing MCPIP3 promoted changes in gene expression patterns similar to those in unstimulated cells (Fig. S4A-B). In the case of *CCNB1*, but not of *KRT14*, *CLCA2* nor *SPRR2D*, the underlying mechanism is dependent on direct binding of MCPIP3 and nucleolytic cleavage within the corresponding 3’UTR sequence (Figs. 6C and Fig. S4C**)**. Finally, we analysed the functional effect of silencing MCPIP3 at later timepoints. The MTT viability test indicated that in HaCaT keratinocytes, 96 h treatment with siRNA has a minor positive effect on their viability (Fig. 6D).

## Discussion

Keratinocytes play key roles in maintaining the protective and immunological functions of the skin. The activation of basal keratinocytes in response to epidermal injury is a key process leading to the activation of proliferation and differentiation processes and to the restoration of the barrier function of the epithelium [34]. We and others previously investigated the role of MCPIP1 RNase in the skin and demonstrated that its activity is mostly important for the regulation of skin immunity [17, 19–21, 35]. This study identified MCPIP3 as a novel factor whose activity in keratinocytes is essential for ensuring the proper balance of epidermal proliferation and differentiation.

Our studies utilizing NHEKs revealed that the expression of MCPIP1 and MCPIP3 is differentially modulated during in vitro differentiation and in response to treatment with tumor-promoting phorbol esters (TPA). We found that the protein level of MCPIP3 was not significantly altered in differentiated keratinocytes in vitro, but its level was rapidly and significantly increased upon treatment with TPA. This phenomenon is in complete opposition to that of MCPIP1, whose expression increases with the induction of keratinocyte differentiation. We correlated the upregulation of MCPIP3 in TPA-treated cells with the activation of PKCδ, which suggests the involvement of PKCδ signaling in the regulation of *ZC3H12C*/MCPIP3 expression. PKCδ belongs to the family of serine/threonine kinases, but unlike classical PKCs (e.g., PKCα), it does not respond directly to the calcium gradient [36]. Importantly, the mechanism of tumor promotion by TPA is mostly explained by its affinity for protein kinase C (PKC) [37]. Aberrant PKC signaling has been implicated in many cellular processes related to carcinogenesis, including cell cycle regulation, apoptosis, differentiation, metastasis and angiogenesis [38]. Thus, the role of MCPIP3 in skin carcinogenesis should be further investigated.

We also demonstrated that the treatment of NHEKs with MCPIP3-specific siRNA led to changes in the transcript levels of a plethora of proliferation- and differentiation-related factors. Significant upregulation of KRT14 and cyclin B1 mRNAs was observed in MCPIP3-silenced cells. This change was accompanied by changes in the transcript levels of many differentiation factors, such as *CLCA2*,* CSTA*,* FLG*,* IVL*, and *S100A7/A9*, further suggesting the functional implication of MCPIP3 RNase in the regulation of epidermal morphology. The limitation of this part of the study was the use of 2D keratinocyte cultures. Because MCPIP3 targets *ZC3H12A*/MCPIP1 mRNA, the silencing of MCPIP3 in our model led to the significant upregulation of the MCPIP1 protein. We and others previously demonstrated that MCPIP1 functions as a suppressor of cell proliferation in many cell types [17, 39]. The effect of MCPIP3 silencing in our 2D model may have been biased by the accumulation of endogenous MCPIP1. Thus, although MCPIP3 negatively regulates the mRNA levels of cyclin B1, a key modulator of cell cycle progression, silencing MCPIP3 had a very minor phenotypic effect on the viability of HaCaT keratinocytes. Thus, to elucidate and clarify the biological function of MCPIP3 in the context of epidermal homeostasis, we developed and characterized in vivo model.

As part of this study, we generated mice with conditional deletion of MCPIP3 in keratinocytes. Although no obvious phenotypic abnormalities were observed upon keratinocyte-specific loss of MCPIP3, we observed substantial changes in the morphology of the epidermis. In particular, a thickened epidermis accompanied by a higher epidermal proliferation rate and impaired stratification were observed. This change was also reflected by the profiling of mRNAs in whole-skin lysates, which revealed significantly increased expression of a plethora of transcripts encoding key modulators of cell cycle progression and nuclear division, such as cyclin E2, cyclin A2, cyclin B1/B2, cdk1 and cdc20. Moreover, compared with those of the controls, the skin of MCPIP3^EKO^ mice was characterized by elevated expression levels of a group of genes that are functionally classified as modulators of epidermis development. These genes included the gene encoding Lgr5, a marker of stemness, and stathmin/Stmn1, whose increased level in the basal epidermis was shown to correlate with the hyperproliferative phenotype of the epidermis and whose activity is important for promoting cutaneous regeneration [40]. We also detected increased mRNA levels of *stefin-3* (*Stfa3*) in the MCPIP3^EKO^ skin. *Stfa3* is an ortholog of the human *CSTA* gene encoding cystatin A, a skin barrier cysteine protease inhibitor that modulates cell adhesion. This molecule is a component of the cornified envelope, and its loss-of-function mutations have been linked to the etiology of peeling skin syndrome [41]. Taken together, the RNA profiles reflected the morphological abnormalities observed in the skin of 12-week-old MCPIP3^EKO^ mice. Importantly, in our in vivo models, the clear abnormalities in MCPIP3^EKO^ skin morphology were not accompanied by excessive inflammation, as in the case of keratinocyte-specific knockout of MCPIP1.

We next investigated whether these discrete morphological abnormalities in MCPIP3^EKO^ skin promote increased responsiveness to TPA and IMQ, which are potent stimulants commonly used to model proliferative and inflammatory disorders. Epidermal hyperplasia caused by keratinocyte-specific loss of MCPIP3 was markedly increased by topical treatment of mouse skin with TPA. An increased proliferation rate promoted the abnormal differentiation of TPA-treated mouse skin. Analysis of the markers of the latest stages of epidermal differentiation revealed increased expression of *Flg* and *Ivl* and accumulation of FLG + cells, indicating disturbances in the composition of the cornified envelope layer. This finding is consistent with our studies utilizing NHEKs, in which treatment with TPA led to a decrease in cell proliferation, an abnormal differentiation pattern, and increased expression of the MCPIP3 protein.

Psoriasis is a chronic, systemic disease that requires dynamic immune responses from both keratinocytes and immune cells. It is a well-characterized disease; however, the exact etiological mechanisms remains to be clarified [42, 43]. IL-23/Th17, IL-36 and IFN signaling are mostly associated with the pathogenesis of this disease. Liu et al. previously demonstrated that MCPIP3 in myeloid cells is important for skin inflammation. Our study contributes to previous knowledge by revealing the role of MCPIP3 in keratinocytes.

Psoriatic lesions display immune cell infiltration and epidermal thickening. In MCPIP3^EKO^ mice, epidermal thickening is separated from excessive inflammation. In the IMQ-induced inflammation model, no difference was observed between the control and MCPIP3^EKO^ mouse skin over the 6-day time course. However, significant differences were observed at the molecular level, suggesting that MCPIP3 activity may be rather important at later time points, including psoriatic relapse. Importantly, patients with similar PASI scores do not always have the same response to the level of treatment effectiveness [44]. Thus, the identification of novel factors important for the regulation of epidermal morphology is crucial for better understanding disease etiology and, ultimately, for the development of novel diagnostic and therapeutic strategies.

Compared with the control mice, the mice with keratinocyte-specific loss of MCPIP3 showed greater responsiveness to stimulation with IMQ, manifested as increased hyperplasia and abnormal differentiation. At the molecular level, the expression of a proliferative marker (KRT14) and a plethora of early and late differentiation markers, including *Krt10*,* Clca2*,* Sprr2d*,* Ivl*,* Lor*, and *S100a9* increased. The protein expression of the skin barrier protein filaggrin was also greater in the IMQ-treated MCPIP3^EKO^ skin than in the control skin. Moreover, in vitro studies on human keratinocytes have shown that the expression of *ZC3H12C*/MCPIP3 is upregulated by treatment with cytokines typically referred to as pro-psoriatic cytokines—IL-17a, IL-36α, and TNF-α [2, 27, 42, 45]. In particular, IL-17a upregulated the expression of MCPIP3 after 0.5 h of stimulation, whereas IL-36α and TNF-α were upregulated after 3 h of stimulation. In IL-36α-stimulated cells, MCPIP3 migrated more slowly according to gel electrophoresis, suggesting that MCPIP3 was post-translationally modified (possibly by phosphorylation). The activation kinetics of MCPIP3 differed from those of MCPIP1. IL-36α led to rapid degradation of MCPIP1 (0.5 h), but at later time points, it resulted in strong upregulation of the protein (3 h). Our results are consistent with those previously reported by Takaishi et al. for MCPIP1 [20] and suggest the involvement of both proteins in the pathology of skin diseases related to the IL-17 and IL-36 axes. We and others previously showed that deletion of MCPIP1 in keratinocytes leads to upregulation of the mRNA levels of IL-36 proteins [17, 20]. On the other hand, IL-36α is a factor stimulating expression of MCPIP3 in keratinocytes, which suggests existence of autostimulatory loop.

Taken together, the results of this study suggest that MCPIP3 may play a role in hyperproliferative skin disorders, including pathologies characterized by an uncontrolled proliferation of keratinocytes, such as psoriasis, skin dermatoses, and eventually nonmelanoma skin cancer (the latter being the subject of our current study). We speculate that in IL-17- and IL36-driven dermatoses, such as psoriasis, the upregulation of MCPIP3 in the keratinocyte compartment could be a mechanism to prevent uncontrolled hyperproliferation. In the future, analysis of MCPIP3 protein expression in psoriatic tissues may shed new light on the involvement of this RNase in the pathogenesis of psoriasis at different stages.

Overall, our in vitro and in vivo analyses revealed that the MCPIP3-driven mechanism controlling the expression of keratinocyte proliferation and differentiation markers overlaps between human and mouse models (Fig. 6E). We found that in vivo, MCPIP3 altered the basal–suprabasal switch. One of the underlying mechanisms involves direct negative regulation of the transcript encoding cyclin B1, a key factor that promotes cell division. As a consequence of enhanced proliferation rate, increased production of keratinocytes changes their subsequent differentiation program.

## Conclusions

Our study identified MCPIP3 as a novel modulator of epidermal biology, whose activity in keratinocytes is one of the mechanisms ensuring their proper proliferation and differentiation equilibrium.

## Electronic supplementary material

Below is the link to the electronic supplementary material.

Supplementary Material 1

Supplementary Material 2

## Data Availability

The RNA sequencing data are available in the NCBI Gene Expression Omnibus under GSE289610. Other data underlying this article will be shared upon reasonable request to the corresponding author.

## Abbreviations

DEG: Differentially expressed gene
IMQ: Imiquimod
KRT: Keratin
NHEKs: Primary normal human epidermal keratinocytes
TPA: 12–O–tetradecanoylphorbol–13–acetate
