# Supplementary material for: MCPIP3 orchestrates the balance of epidermal proliferation and differentiation
Source: Cell Commun Signal. 2025 Apr 8;23:175. doi: 10.1186/s12964-025-02184-1 (PMC11980240; doi:10.1186/s12964-025-02184-1)
Supplement: Supplementary file 1 — Supplementary Material 1 [file 12964_2025_2184_MOESM1_ESM.docx]

**Supplementary Tables**

**Table S1.** List of primers used in the study.

|  | |  | **Forward (5’->3’)** | **Reverse (5’->3’)** |
| --- | --- | --- | --- | --- |
| **Primers used for genotyping** | | **loxP** | GTATGCCTGTTTGCCTCAAATTTCATG | TGGCCAGTGAGGAATGGAATAACA |
|  |  | **Krt14 Cre** | GATGAAAGCCAAGGGGAATG | CATCACTCGTTGCATCGACC |
| **Primers used for cloning** | | ***CCNB1-3’UTR*** | ATGCTAGCgttcaagatttagccaaggctgtgg | ATGTCGACccaaaacacaaaaccaaaatgaaaactggc |
|  |  | ***KRT14- 3’UTR*** | ATGCTAGCggctgcccagcc | ATGTCGACttatgcaactcagataatgaagct |
|  |  | ***SPRR2D-3’UTR*** | ATGCTAGCagagcaagtaacagcttcag | ATGTCGACtatatgcatagatactttattcagggag |
|  |  | ***CLCA2-3’UTR*** | ATGCTAGCgcagacaagaaagagaatggaac | ATGTCGACggcaaatgattttattgttcgataatc |
| **Primers used for RT-qPCR** |  | ***Ef2/EF2*** | GACATCACCAAGGGTGTGCAG | TCAGCACACTGGCATAGAGGC |
|  | **Murine genes** | ***Ccna2*** | CTCGAGGCATTCGGGTCG | TAAGAGGAGCAACCCGTCG |
|  |  | ***Ccnb1*** | GGCCTCACAAAGCACATGACTG | GCCTAAACTCAGAAGCAACAACAT |
|  |  | ***Ccnb2*** | ATGTCAACAAGCAGCCGAAAC | TCAGAGAAAGCTTGGCAGAGG |
|  |  | ***Ccne2*** | GGAGGAATCAGCCCTTGCAT | CCTGTGAACATGCCCAGCTTA |
|  |  | ***Cdc20*** | CGCCCACCAAAAAGGAGCAT | GATTTCGGGGGCATCAAGGA |
|  |  | ***Cdk1*** | ATTGTGTTTTGCCACTCCCG | GCGTCACTACCTCGTGTGTGTA |
|  |  | ***Ckap2*** | CACGATTGCAGGAGCAAGTG | ACTCCGTCATCTGAGCTTTCC |
|  |  | ***Clca2*** | ACCAGGTGTTCATCTGACATTAC | TACAGAATTCAACCACAGAAGGT |
|  |  | ***Flg*** | GGAGGCATGGTGGAACTGA | TGTTTATCTTTTCCCTCACTTCTACATC |
|  |  | ***Il1b*** | TGCCACCTTTTGACAGTGATG | TGATGTGCTGCTGCGAGATT |
|  |  | ***Il1f6*** | ACTAAGAGCAGCATCACCTTCG | CCAGATATTGGCATGGGAGCAA |
|  |  | ***Il1f9*** | AGTTCCACGAAGCCACAGAGTA | TAGCAGCAAAGTAGGGTGTCCA |
|  |  | ***Il6*** | ACTTCACAAGTCGGAGGCTT | GGTACTCCAGAAGACCAGAGG |
|  |  | ***Il17a*** | ACTACCTCAACCGTTCCACG | TTCCCTCCGCATTGACACAG |
|  |  | ***Il22*** | AGACAGGTTCCAGCCCTACA | CCAGTTCCCCAATCGCCTT |
|  |  | ***Ivl*** | TCTCCCTCCTGTGAGTTTGTTTG | TGTGGAGTTGGTTGCTTTGCT |
|  |  | ***Krt7*** | GATTGCGGAGATGAACCGCT | CTAACTTGGCACGCTGGTTCT |
|  |  | ***Krt10*** | TCAAGGAGTGGTACGAGAAGCA | GTTGTCAGGGTGAGGATCTGC |
|  |  | ***Krt14*** | TCCTGCTGGATGTGAAGACAAG | GCACATCCATGACCTTGGTGC |
|  |  | ***Lcn2*** | GAACTTGATCCCTGCCCCAT | TTCTGATCCAGTAGCGACAGC |
|  |  | ***Lgr5*** | GGGAACCGAGCCTTACAGAG | AAGGTCCCGCTCATCTTGAA |
|  |  | ***Lor*** | ACTCATCTTCCCTGGTGCTTC | CTTTCCACAACCCACAGGAG |
|  |  | ***S100a9*** | CATGGAGGACCTGGACACAAAC | CCCTTTAGACTTGGTTGGGCAG |
|  |  | ***Sprr2d*** | GCCCACCTAAGAGCAAGTGAG | GGAGGGTGAAAGGTGAAGGAGA |
|  |  | ***Stmn1*** | CGCTTGCGAGAGAAGGACAAG | TATTTAGGACGGGGTCGGGG |
|  |  | ***Stfa3*** | AATAAAGGGAGGCCTGTCAGAG | GTCCAGCAACGACTTGAGATTT |
|  |  | ***Zc3h12c*** | ATGCGAGAAACCTCATCCCTG | TCCAAAAACCAATCCACCGC |
|  | **Human genes** | ***CCNB1*** | CCTCTCCAAGCCCAATGGAA | ACTTCCCGACCCAGTAGGTA |
|  |  | ***CLCA2*** | ACTGTGGGCAACGACACTAT | GTCCAGTGCCCAGGCTTAG |
|  |  | ***CSTA*** | CCTGGAGGCTTATCTGAGGC | TGCACAGCTTCCAATTTTCCG |
|  |  | ***FLG*** | AAGGTTCACATTTATTGCCAAA | GGATTTGCCGAAATTCCTTT |
|  |  | ***IVL*** | GATGTCCCAGCAACACACAC | TGCTCACATTCTTGCTCAGG |
|  |  | ***KRT1*** | ATTTCTGAGCTGAATCGTGTGATC | CTTGGCATCCTTGAGGGCATT |
|  |  | ***KRT10*** | AGTCCCAACTGGCCTTGAAAC | TGCACACAGTAGCGACCTTC |
|  |  | ***KRT14*** | CCAGCTCAGCATGAAAGCATC | TGAGATCCAGAGGAGAACTG |
|  |  | ***S100A7*** | AAGAAAGATGAGCAACAC | CCAGCAAGGACAGAAACT |
|  |  | ***S100A9*** | GCTCCTCGGCTTTGACAGAGTGCAAG | GCATTTGTGTCCAGGTCCTCCATGATGTGT |
|  |  | ***SPRR2D*** | TGCATCTTCTCACCAAAGCCT | ACAGCTGAGGACTTCCTTTTCTT |
|  |  | ***ZC3H12C*** | AGAGGTCTGAATCTCCAATGCAA | CGGGATTGCTCTTTCCTCCAA |

**Table S2.** List of antibodies used for western blot (WB) or IHC analysis.

| **Antigen** | **Host species** | **Working dilution (application)** | **Catalogue number** | **Company** |
| --- | --- | --- | --- | --- |
| **MCPIP1** | Rabbit | 1:2000 (WB) |  | Own production |
| **MCPIP3** | Rabbit | 1:1000 (WB) | GTX85195 | GeneTex Irvine, CA, USA |
| **Filaggrin** | Rabbit | 1:200 (IF) | 905804 | BioLegend, San Diego, CA, USA |
| **Keratin 10** | Rabbit | 1:200 (IF) | ab76318 | Abcam, Cambridge, UK |
| **Keratin 14** | Mouse | 1:200 (IF) | ab7800 | Abcam, Cambridge, UK |
| **PCNA** | Mouse | 1:200 (IF) | CBL407 | Sigma Aldrich |
| **F4/80** | Rat | 1:200 (IF) | 11-4801-81 | eBioscience, Frankfurt am Main, Germany |
| **Phospho-PKCα/β II (Thr638/641)** | Rabbit | 1:1000 (WB) | 9375 | Cell Signaling Technology, Danvers, MA, USA |
| **Phospho-PKC**δ **(Thr505)** | Rabbit | 1:1000 (WB) | 9374 | Cell Signaling Technology |
| **α-tubulin** | Mouse | 1:1000 (WB) | CP06 | Calbiochem, San Diego, USA |
| **rabbit IgG, HRP-linked** | Goat | 1:20000 | A0545 | Sigma Aldrich |
| **mouse IgG, HRP-linked** | Goat | 1:20000 | 554002 | Pharmingen, San Diego, CA, USA |
| **Alexa Fluor 488 anti-mouse** | Goat | 1:500 (IF) | A11008 | Invitrogen, Darmstadt, Germany |
| **Alexa Fluor 546** **anti-rabbit** | Goat | 1:500 (IF) | A11035 | Invitrogen, Darmstadt, Germany |
| **Alexa Fluor 594 anti-rat** | Goat | 1:500 (IF) | A11007 | Invitrogen, Darmstadt, Germany |
