# Supplementary material for: MCPIP3 orchestrates the balance of epidermal proliferation and differentiation
Source: Cell Commun Signal. 2025 Apr 8;23:175. doi: 10.1186/s12964-025-02184-1 (PMC11980240; doi:10.1186/s12964-025-02184-1)
Supplement: Supplementary file 2 — Supplementary Material 2 [file 12964_2025_2184_MOESM2_ESM.docx]

**
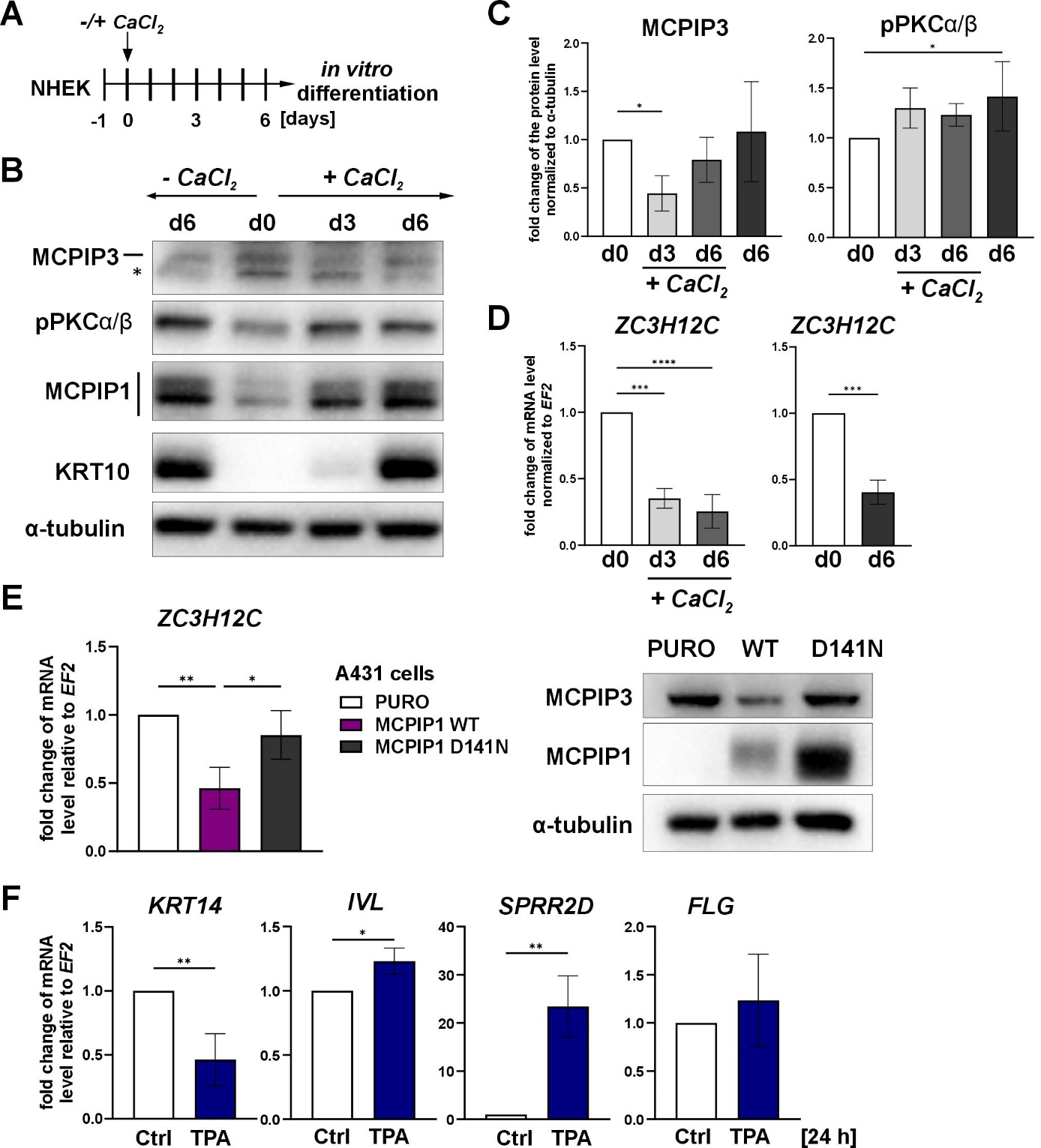
**

**Figure S1. (A)** An outline of the protocol for *in vitro* differentiation. **(B)** Western blot analysis of MCPIP1, MCPIP3, p-PKCα/β II (Thr638/641)**,** and α-tubulin in NHEK cells during *in vitro* differentiation; * indicates an unspecific band. **(C)** Densitometric analysis of protein bands from western blot analysis (n=3-5). **(D)** RT‒qPCR analysis of *ZC3H12C* transcript levels in NHEK cells during *in vitro* differentiation (n=3). **(E)** A431 pLIX PURO, MCPIP1 WT, or MCPIP1 D141N cells were stimulated with doxycycline for 18h. Graph represents the results of RT-qPCR analysis of *ZC3H12C* transcript levels (n=3). Western blot indicates expression level of MCPIP1, MCPIP3 and α-tubulin. **(F)** RT‒qPCR analysis of *KRT14, IVL, SPRR2D* and *FLG* transcript levels in NHEKs treated with TPA for 24 h (n=3-4). The data are shown as the means ± standard deviations. *EF2* was used as a reference gene (D, F). One-way ANOVA (C, D, E) or unpaired t test (D, F) was used for statistical analysis; * p<0.05, ** p<0.01, *** p < 0.001, and ****p<0.0001.


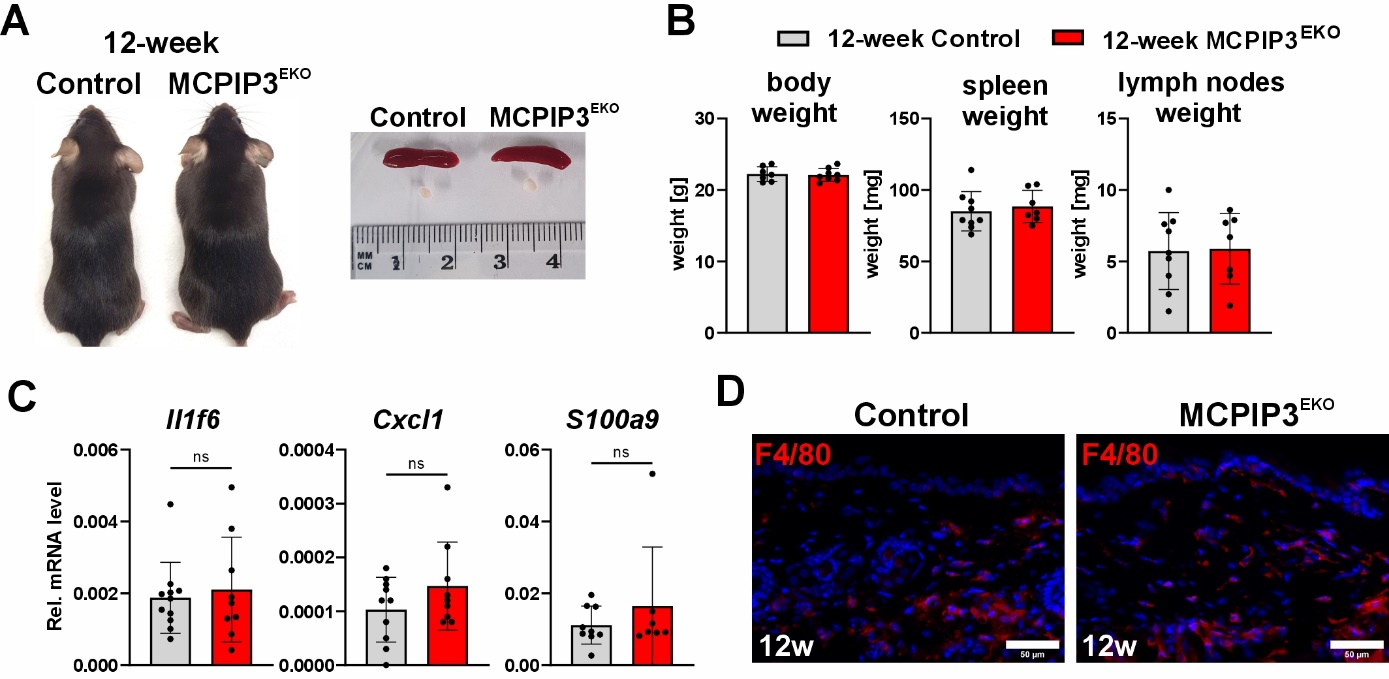


**Figure S2. (A)** Macroscopic appearance of control and MCPIP3^EKO^ littermate mice and their spleens and lymph nodes at 12 weeks of age. **(B)** Female body weights at 12-weeks (n=7-8) and spleen and lymph node weights at 12 weeks (n=7-9). The data are shown as the means ± standard deviations. (**C)** RT‒qPCR analysis of *Il1f6*, *Cxcl1*, and *S100a9* expression levels in the skin of 12-week-old control and MCPIP3^EKO^ mice skin (n=7-11). (**D)** Representative F4/80 staining of 12-week-old control and MCPIP3^EKO^ mice skin. The data are shown as the means ± standard deviations. *Ef2* was used as a reference gene. An unpaired t test was used for statistical analysis; ns p > 0.01.

**
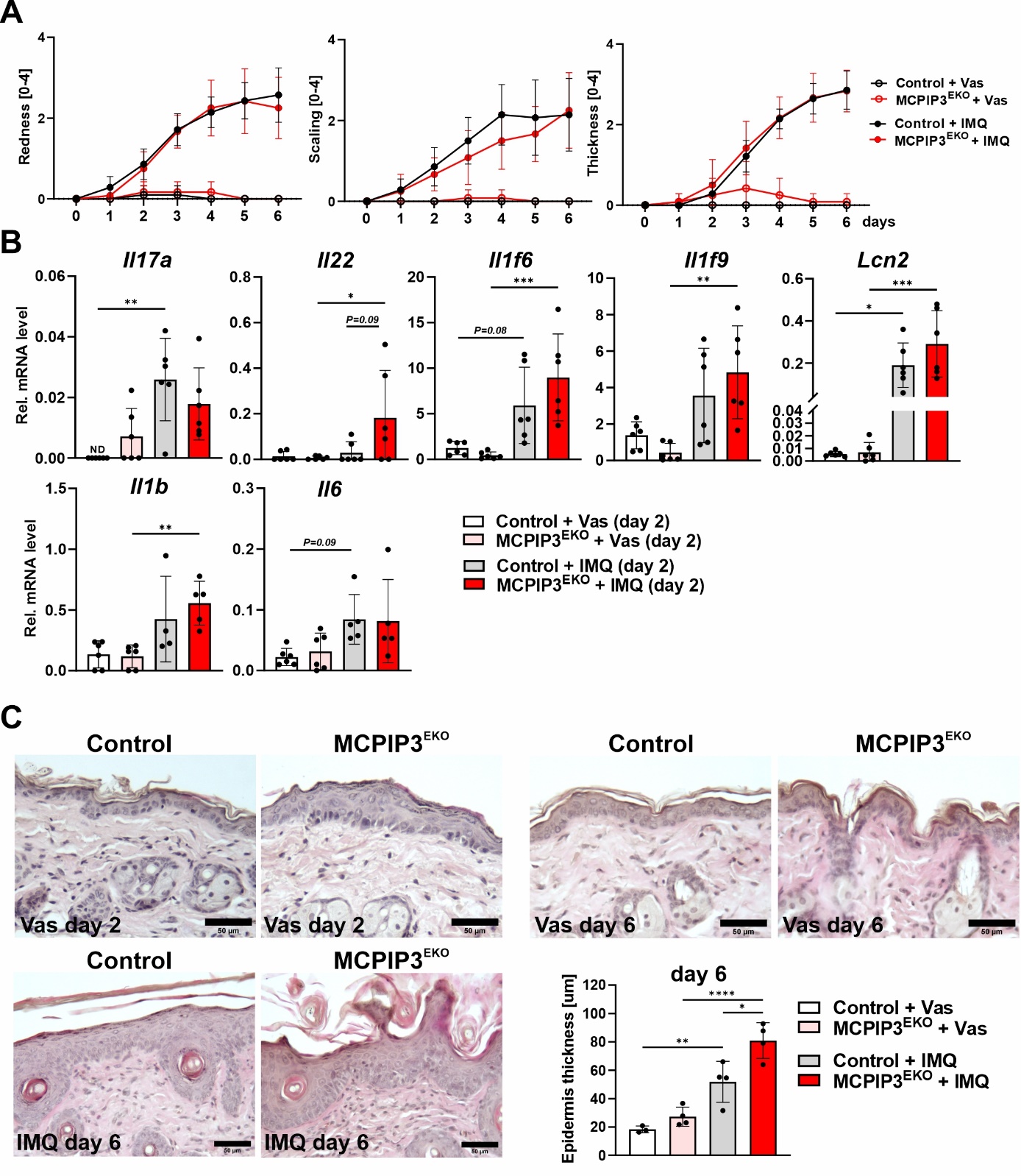
**

**Figure S3. (A)** Graphs showing the PASI scores for erythrema, scaling and thickness over the 6 days of treatment (n=5–7). **(B)** RT–qPCR analysis of *Il17a, Il22, Il1f6, Il1f9, Lcn2, Il1b* and *Il6* expression levels in control and MCPIP3^EKO^ skin treated with Vaseline or IMQ for 2 days (n=4–6). *Ef2* was used as a reference gene. **(C)** Representative H&E staining of Vaseline-treated mouse skin on days 2 and 6, and that of IMQ-treated mice on day 6. The graph shows the quantification of epidermal thickness on day 6 (n=3–4). The data are shown as the means ± standard deviations. One-way ANOVA was used for statistical analysis; * p < 0.05, ** p < 0.01, and *** p < 0.001.

**
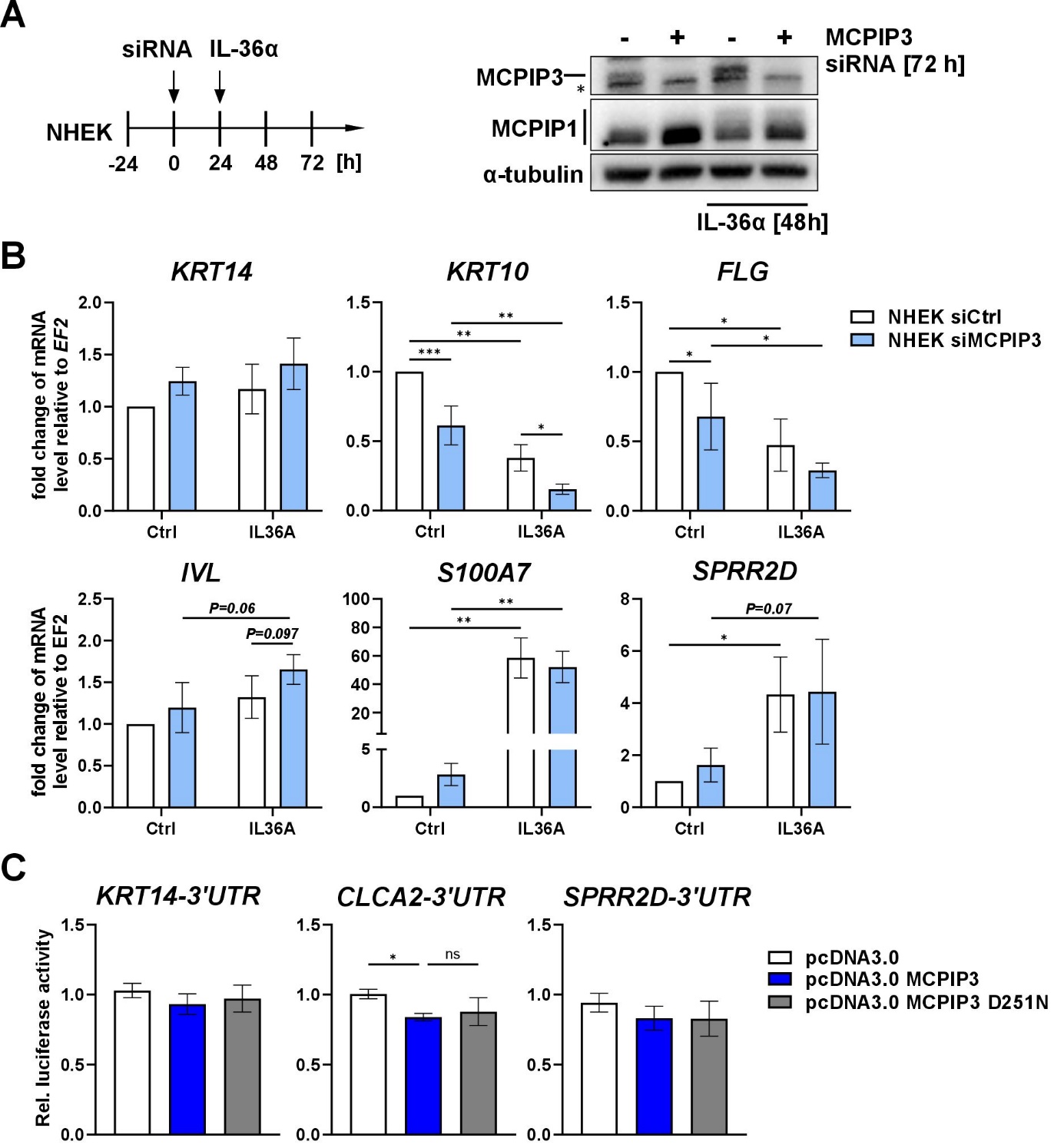
**

**Figure S4. (A-B)** NHEK cells were treated with control or MCPIP3 siRNA for 24 h and then stimulated with or without IL-36α for 48 h. Cells were analysed 72 h after siRNA treatment. (A) Western blot analysis of MCPIP1, MCPIP3 and α-tubulin; * indicates an unspecific band. (B) RT‒qPCR analysis of *KRT14, KRT10, FLG, IVL, S100A7,* and *SPRR2D* expression levels (n=3). *Ef2* was used as a reference gene. **(C)** HEK293 cells were cotransfected with a luciferase reporter pmirGLO plasmid containing the 3'UTR of human *KRT14*, *CLCA2* or *SPRR2D* and pcDNA3.0 (empty) or pcDNA3.0 encoding MCPIP3 or a catalytically inactive D251N mutant. The graphs show the calculated luciferase activity normalized to that of the pmirGLO-empty vector (n=4-5). The data are shown as the means ± standard deviations. Two-way ANOVA (B) or one-way ANOVA (C) was used for statistical analysis; * p < 0.05, ** p < 0.01, and *** p < 0.001; ns p>0.1.
